# Gut Microbiota Profile and Its Association with Clinical Variables and Dietary Intake in Overweight/Obese and Lean Subjects: A Cross-Sectional Study

**DOI:** 10.3390/nu13062032

**Published:** 2021-06-13

**Authors:** Judit Companys, Maria José Gosalbes, Laura Pla-Pagà, Lorena Calderón-Pérez, Elisabet Llauradó, Anna Pedret, Rosa Maria Valls, Nuria Jiménez-Hernández, Berner Andrée Sandoval-Ramirez, Josep Maria del Bas, Antoni Caimari, Laura Rubió, Rosa Solà

**Affiliations:** 1 Eurecat, Centre Tecnològic de Catalunya, Unitat de Nutrició i Salut, 43204 Reus, Spain; judit.companys@eurecat.org (J.C.); laura.pla@eurecat.org (L.P.-P.); lorena.calderon@eurecat.org (L.C.-P.); josep.delbas@eurecat.org (J.M.d.B.); rosa.sola@urv.cat (R.S.); 2 Functional Nutrition, Oxidation, and Cardiovascular Diseases Group (NFOC-Salut), Facultat de Medicina i Ciències de la Salut, Universitat Rovira i Virgili, C/Sant Llorenç 21, 43201 Reus, Spain; elisabet.llaurado@urv.cat (E.L.); andreesandoval1@gmail.com (B.A.S.-R.); 3 Fundación para el Fomento de la Investigación Sanitaria y Biomédica, 46020 Valencia, Spain; nuria.jimenez@uv.es; 4 CIBER en Epidemiología y Salud Pública, 28029 Madrid, Spain; 5 Eurecat, Centre Tecnològic de Catalunya, Biotechnology Area, 43204 Reus, Spain; antoni.caimari@eurecat.org; 6 XaRTA-TPV, Agrotecnio Center, Escola Tècnica Superior d’Enginyeria Agrària, Food Technology Department, University of Lleida, Avda/Alcalde Rovira Roure 191, Lleida, 25198 Catalonia, Spain; laura.rubio@udl.cat; 7 Hospital Universitari Sant Joan de Reus, 43201 Reus, Spain

**Keywords:** obesity, gut microbiota, dietary intake, saturated fatty acids

## Abstract

We aimed to differentiate gut microbiota composition of overweight/obese and lean subjects and to determine its association with clinical variables and dietary intake. A cross-sectional study was performed with 96 overweight/obese subjects and 32 lean subjects. Anthropometric parameters were positively associated with *Collinsella aerofaciens*, *Dorea formicigenerans* and *Dorea longicatena*, which had higher abundance the overweight/obese subjects. Moreover, different genera of *Lachnospiraceae* were negatively associated with body fat, LDL and total cholesterol. Saturated fatty acids (SFAs) were negatively associated with the genus *Intestinimonas*, a biomarker of the overweight/obese group, whereas SFAs were positively associated with *Roseburia*, a biomarker for the lean group. In conclusion, *Dorea formicigenerans*, *Dorea longicatena* and *Collinsella aerofaciens* could be considered obesity biomarkers, Lachnospiraceae is associated with lipid cardiovascular risk factors. SFAs exhibited opposite association profiles with butyrate-producing bacteria depending on the BMI. Thus, the relationship between diet and microbiota opens new tools for the management of obesity.

## 1. Introduction

The worldwide prevalence of obesity has nearly tripled since 1975 [1]. Age, family history, genetics, sex, race and ethnicity are all unmodifiable risk factors that influence the occurrence of obesity, whereas the diet represents the main modifiable risk factor for tackling the incidence of obesity [2].

Specific food groups or certain dietary compounds, such as the high consumption of red meat, saturated fatty acids (SFAs) and the insufficient intake of fiber, have been linked to the onset and progression of obesity [3,4,5].

An approach for the treatment of obesity developed in recent years involves a study of the gut microbiota, which results in a description of the significant differences between the gut microbiota of lean subjects and that of obese subjects. For example, an increased Bacteroidetes/Firmicutes ratio in the gut has been associated with obesity development in obese subjects [6]. Moreover, a lower microbial diversity has been observed in obese subjects compared with lean subjects [6]. In this context, few anthropometric variables, such as visceral fat accumulation and waist circumference (WC), are related to the gut microbiota. For instance, an inverse association between the presence of the genus *Blautia* and visceral fat accumulation has been found in Japanese adults [7], whereas a positive association has been found between the presence of the genus *Acidaminococcus* and the body mass index (BMI), WC and hip circumferences in Bangladeshi adults [8].

Additionally, dietary intake is considered a modulator of the gut microbiota in obese subjects. For instance, a high intake of fat and SFAs, as observed in randomized controlled trials, can reduce the richness and diversity of the gut microbiota [5], whereas a high intake of proteins, as observed in observational studies, is correlated with high concentrations of Bacteroidetes, a phylum related to weight loss, in the gut microbiome [9].

Even though the available evidence highlights the characteristics of the gut microbiota in obese subjects, information about the association between clinical variables linked to obesity and dietary intake and the gut microbiota in humans remains scarce.

The hypothesis investigated in the present study is that clinical variables and dietary intake are associated with different gut microbiota compositions in both overweight/obese and lean subjects. Therefore, in the present cross-sectional study, we aimed to differentiate the gut microbiota of overweight/obese from that of lean subjects, and to determine its association with clinical variables and dietary intake.

## 2. Materials and Methods

### 2.1. Study Design and Participants

A cross-sectional study was performed with two different populations: (1) 96 overweight/obese (body mass index (BMI) ≥ 25 kg/m^2^ and ≤35 kg/m^2^) subjects and (2) 32 lean subjects (BMI < 25 kg/m^2^). All subjects were recruited between June 2016 and December 2018 from Reus and its outskirts (Spain), and all study visits took place in Eurecat, Centre Tecnològic de Catalunya, Unitat de Nutrició i Salut.

The inclusion criteria were as follows: (1) obese group, >18 years of age, abdominal obesity (WC ≥ 88 cm in women and ≥102 cm in men) and signed informed consent; and (2) lean group, >18 years of age, BMI ≤ 25 kg/m^2^ and signed informed consent.

The exclusion criteria were the following: use of antibiotics in the 30 days prior to the study; subjects suffering from diabetes, gastrointestinal chronic disease or anemia (hemoglobin: <13 g/dL in men; <12 g/dL in women); BMI < 18 kg/m^2^ and >40 kg/m^2^; women pregnant or breastfeeding; subjects who recently followed a hypocaloric diet or weight loss pharmacotherapy treatment; and subjects who participated in a clinical trial or nutritional intervention study in the 30 days before inclusion in the study.

All subjects signed an informed consent form prior to their participation in the study, which was approved before the start of the study period by the Clinical Research Ethical Committee of HUSJ, Reus, Spain. The protocols and trials were conducted in accordance with the Helsinki Declaration and Good Clinical Practice Guidelines of the International Conference of Harmonization (ICH GCP) and reported as STROBE-nut: An extension of the STROBE statement for nutritional epidemiology [10].

### 2.2. Clinical Parameters

#### 2.2.1. Anthropometric Measurements

All parameters were measured by trained dietitians at the baseline visit. Anthropometric data were obtained with the subjects wearing no shoes. The WC was measured at the umbilicus using a 150-cm anthropometric steel measuring tape. The body weight, fat mass, lean mass, muscle mass, bone mass and total water were measured with a TANITA SC 330 S portable scale (Peroxfarma, Barcelona, Spain). The height was measured with a Tanita Leicester Portable (Tanita Corp., Barcelona, Spain), and the BMI was calculated as the ratio of the measured weight (kg) to the square of the height (m).

#### 2.2.2. Blood Pressure Measurements

At the baseline visit, the systolic blood pressure (SBP) and diastolic blood pressure (DBP) were measured by trained physicians twice, at 1 minute intervals between measurements. The mean blood pressure values were recorded, using an automatic sphygmomanometer (OMRON HEM-907; Peroxfarma, Barcelona, Spain) while with the volunteers in a seated position.

#### 2.2.3. Lipid Profile and Glucose Measurement

The total cholesterol, high-density lipoprotein (HDL) cholesterol, triglycerides and glucose concentrations in serum were measured using by standardized enzymatic automated methods with an autoanalyzer (Beckman Coulter-Synchron, Galway, Ireland) at the baseline visit. The low-density lipoprotein (LDL) cholesterol level was calculated with the Friedewald formula.

### 2.3. Dietary Intake 

A three-day dietary intake record was recorded by the volunteers within 1 week before the study visit (including two workdays and one weekend day). The volunteers were given instructions on how to record their dietary intake. At the baseline visit, dietitians checked the dietary record along with the volunteers and to checked for missing quantities of products using PortBook to complete any missing information [11]. The mean daily energy and nutrient intakes were calculated from Spanish Food Composition Tables using computerized software (PCN Pro 1.0).

### 2.4. Faecal Sample Collection

At the baseline visit, volunteers received a ProtocultTM stool collection device (ABC, Minnesota, EEUU) to provide a fecal sample. The subjects were asked to collect the fecal sample no later than 72 h before the baseline visit in a sterile pot with 10 mL of RNAlater storage solution (Sigma-Aldrich Quimica SL; Madrid, Spain) and were instructed to subsequently store the sample in the freezer until the baseline visit. After delivery to the investigators, the stool samples were immediately stored at −80 °C until DNA extraction.

### 2.5. Sample Size

The sample size was estimated based on a study [12], who found significant differences in the gut microbiota composition between three lean subjects and four obese subjects, and by another study [13], who found significant differences were observed in the gut microbiota composition between 27 lean subjects and 26 overweight/obese subjects.

Thus, we assumed that an expanded sample size of 32 subjects in the lean group and 96 in the obese group was sufficient to achieve significant power.

### 2.6. Faecal Microbiota Analysis

#### 2.6.1. DNA Purification and Sequencing

The fecal samples stored in RNAlater^®^ were diluted with PBS solution (1:2 dilution) and centrifuged at 2000 rpm and 4 °C for 5 min. A robotic workstation, MagNA Pure LC Instrument (Roche), was used according to the manufacturer’s instructions for the extraction of total DNA from pelleted bacterial cells with the MagNA Pure LC DNA isolation kit III (Bacteria, Fungi) (Roche). The V3-V4 region of the 16S rRNA gene was amplified by PCR with the primers: the forward primer (5’-TCGT CGGC AGCG TCAG ATGT GTAT AAGA GACA GCCT ACGG GNGG CWGCAG-3’) and reverse primer (5’-GTCT CGTG GGCT CGGA GATG TGTA TAAG AGAC AGGA CTAC HVGG GTAT CTAA TCC-3’). This region was used for amplicon library construction according to the Illumina instructions. Sequencing was performed with Kit V3 (2 × 300 cycles) on a MiSeq platform (Illumina, Eindhoven, Netherlands) at the Centre for Public Health Research (FISABIO-Salud Pública, Valencia, Spain). All the sequences have been deposited in the EBI database under the accession numbers PRJEB36385 and PRJEB32411.

#### 2.6.2. Fecal Microbiota Analysis: Sequence Analysis

We applied Prinseq (v0.20.4) [14] for trimming the ends of each read with bases with a quality lower than 30 and discarding reads shorter than 100 bases. The following steps were performed with R (v3.6.0) [15] using the corresponding functions of the DADA2 library (v1.8.0) [16]. Dereplication was performed to combine all identical reads into unique sequences with an abundance equal to the number of reads combined. Based on the dereplicated reads and error estimations, amplicon sequence variants (ASVs) were inferred. The ASVs were aligned using Bowtie2 against the human genome (GRCh38.p11) and matches were subsequently discarded [17]. Taxonomic annotation of ASVs were obtained using the Silva database (version 123). To assign a unique species to each ASV sequence, we searched for 100% similarity matches. Additionally, ASVs with an assigned genus but without exact matching at the species level were aligned with BLAST [18] using the same reference database with a minimum identity of 97%.

Alpha diversity parameters, the Shannon diversity index and the Chao1 richness estimator were calculated using the vegan library in the R package. The structural differences between communities (beta diversity) were assessed by principal coordinates analysis (PCoA) based on the Bray-Curtis dissimilarity index. To determine the contribution of an environmental factor to the variability in the microbiota composition between groups, we performed the ADONIS test using the R package.

### 2.7. Statistical Analysis

Descriptive data are expressed as the means ± standard deviations (SDs), and categorical data are expressed as percentages. Statistical significance was defined as a *p* value < 0.05.

Statistical analyses of the clinical baseline characteristics and comparisons between groups were performed using IBM SPSS Statistics software version 23.0 (SPSS Inc., Chicago, IL, USA).

The normality of the variables was assessed using the Kolmogorov-Smirnov and Shapiro Wilk tests. Student’s t-test was used for the comparisons of normally distributed variables, and the Wilcoxon rank-sum test was used for nonnormally distributed variables. Differences in categorical variables were examined by chi-squared analysis. Differences in continuous variables with a parametric and nonparametric distributions were examined using Student’s t-test and the Mann-Whitney U test, respectively.

A linear discriminant analysis (LDA) effect size (LEfSe) algorithm was used for the detection of ASV biomarkers. The α-value was fixed to <0.05, and the threshold used to consider a discriminative feature for the logarithmic LDA score was set to >2.5 or >3.0. We also performed a Wilcoxon rank-sum test with the compositional data of the overweight/obese and lean groups.

The associations between energy, dietary nutrients or clinical variables and the microbiota composition were evaluated based on ASV biomarkers that exhibited significant differential abundance based on a sparse partial least square (sPLS) analysis, which is a multivariant method described by Best and Roberts (1975) [19] and implemented in the ‘mixOmics’ R package [20]. We used sPLS with ncomp = 3 and 20 variables (for clinical data association) or 30 variables (for diet associations) per component (KeepX) and applied a canonical mode because this method models bidirectional (no causal) relationships between two datasets. The inner product of the coordinates of each variable approximates their association score. This threshold was set to 0.5 to represent the relationships in the networks.

## 3. Results

### 3.1. Baseline Characteristics of the Participants

In this case, 128 subjects were enrolled in the present cross-sectional study. The baseline characteristics of the subjects are presented in Table 1. 

Differences in categorical variables were examined using chi-squared analysis and presented as percentage (%). Differences in continuous variables were examined using T-Student for parametric variables and U the Mann-Whitney for non-parametric variables. Statistical significance was set at *p* value < 0.05.

The overweight/obese group consisted of 96 subjects (61.5% men and 38.5% women) with a mean age of 52.2 ± 9.7 years. The lean group consisted of 32 subjects (50% men and 50% women) with a mean (± SD) age of 40.2 ± 8.9 years. No significant differences in gender were observed among the groups. Even though there were significant differences in age between the two groups, the overweight/obese group was older than the lean group (*p* < 0.001), both groups were in the same range of age (adulthood) and no adjustments were performed, since it has been shown that subjects between 40 and 59 years (middle age) have similar gut microbiota compared with young adults or older adults [21].

The overweight/obese group presented significantly (*p* < 0.001) higher levels of various anthropometric parameters than the lean group: BMI, 31.2 ± 3.4 vs. 23.9 ± 2.6 kg/m^2^; WC, women, 99.3 ± 7.6 vs. 80.5 ± 9.7 cm and men, 111.2 ± 8.1 vs. 87.7 ± 6.6 cm; fat mass, 34.9 ± 8.3 vs. 22.3 ± 8.1%; and total water, 46.6 ± 6.4 vs. 55.1 ± 8.5%. No significant differences in the lean mass, muscle mass or bone mass were observed between the groups.

Moreover, the overweight/obese group presented higher levels of SBP (129.8 ± 15.9 vs. 109.7 ± 7.1 mm Hg), DBP (81.1 ± 9.6 vs. 65.8 ± 5.9 mm Hg), fasting glucose (94.5 ± 10.2 vs. 80.3 ± 7.2 mg/dL), total cholesterol (205.1 ± 30.8 vs. 179.6 ± 34.6 mg/dL), LDL cholesterol (127.4 ± 26.3 vs. 99.4 ± 33.5 mg/dL), HDL cholesterol (52.7 ± 13.0 vs. 63.1 ± 17.6 mg/dL) and triglycerides (125.3 ± 56.8 vs. 85.5 ± 43.0 mg/dL) than the lean group (*p* < 0.001).

No significant differences in physical activity were found between the groups.

### 3.2. Dietary Assessment

The dietary intake characteristics of the subjects based on their 3-day dietary record are presented in Table 2.

Both groups presented a similar average intake with no significant differences in the total energy intake and the intake of protein, total fat, SFAs, monounsaturated fatty acids, polyunsaturated fatty acids (PUFAs), alcohol, dietary cholesterol, sodium and calcium. However, the overweight/obese group consumed a lower daily average of total carbohydrates (mean ± SD) 206.0 ± 53.1 vs. 181.2 ± 60.9 g; *p* = 0.005, simple carbohydrates 92.1 ± 35.9 vs. 78.3 ± 35.8 g; *p* = 0.014, complex carbohydrates 113.8 ± 24.8 vs. 103.8 ± 41.9 g; *p* = 0.017, fibre 23.7 ± 9.1 vs. 18.8 ± 6.8 g; *p* < 0.001, potassium 3042.4 ± 868.5 vs. 3448.3 ± 826.2 mg; *p* = 0.002 and magnesium 309.5 ± 106.9 vs. 345.2 ± 74.0 mg; *p* = 0.001 than the lean group.

### 3.3. Analysis of the Gut Microbiota Composition

Comparisons between the groups at the phylum level were performed (Supplementary Figure S1). The two major phyla in both the overweight/obese and lean groups were Firmicutes (60.44% and 57.61%, respectively) and Bacteroidetes (29.93% and 35.23%, respectively). The overweight/obese group had significantly higher abundances of the phyla Actinobacteria (Bacteria), Firmicutes (Bacteria) and Euryarchaeota (Archaea) (*p* = 5.5 × 10 ^−5^, *p* = 0.056 and *p* = 0.00091, respectively) and significantly lower abundances of the phyla Tenericutes, Lentisphaerae and Bacteroidetes (*p* = 0.00096, *p* = 0.013 and *p* = 0.021, respectively) than the lean group. As a result, the Firmicutes/Bacteroidetes ratio in the overweight/obese group was higher than that in the lean group.

**Figure 1:**
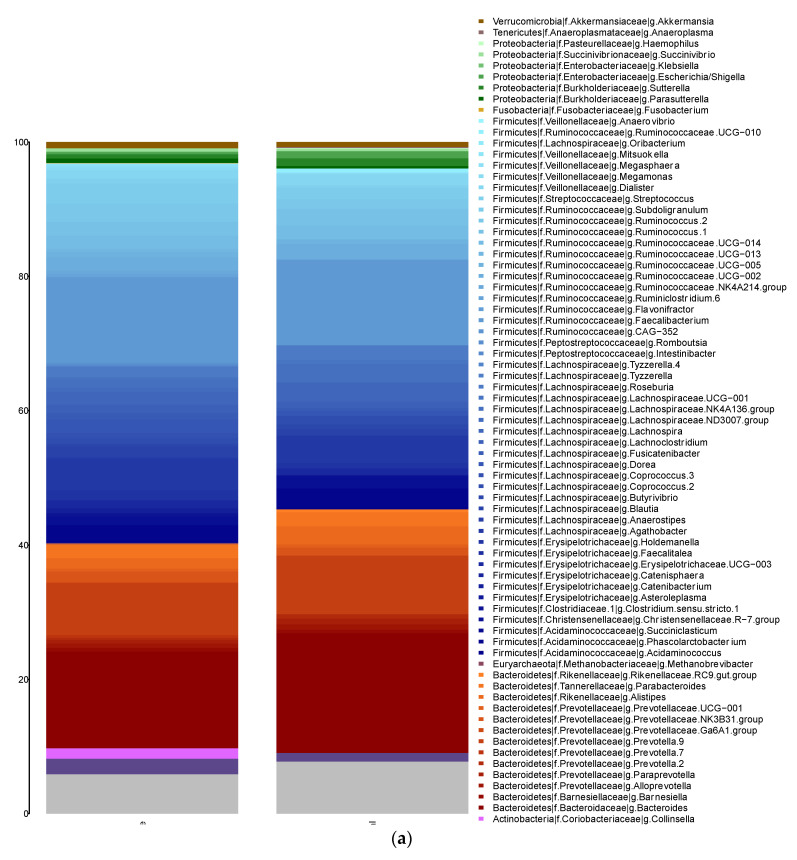

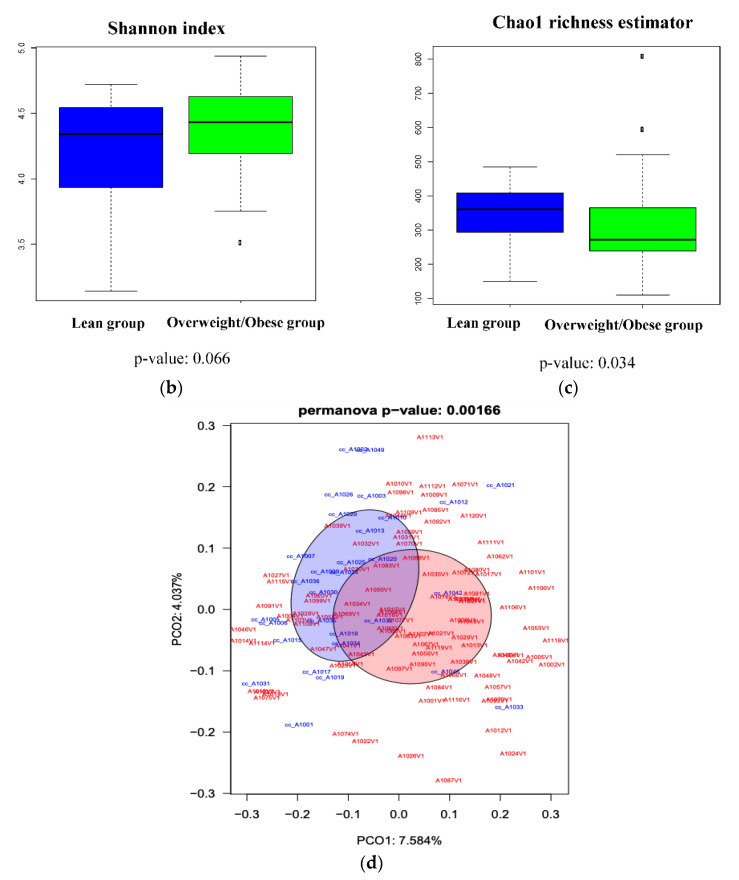
(**a**) Taxonomic composition at the genus level found for each group. The *X*-axis represents the group (overweight/obese and lean groups), and the *y*-axis represents the relative abundance assigned to each genus. (**b**) Differences in diversity (Shannon index) between the obese and lean groups. (**c**) Differences in richness (Chao 1 richness estimator) between the overweight/obese and lean groups. (**d**) Difference in the distribution and variability of the microbiota structure determined by principal coordinates analysis (PCoA) based on the Bray-Curtis dissimilarity index.

Taxonomic assignment was performed at genus level (Figure 1a). The main genera in the overweight/obese and lean groups were the following: a) Firmicutes phylum, *Faecalibacterium* (12.64% and 12.78%, respectively), *Agathobacter* (4.72% and 4.08%, respectively), *Subdoligranulum* (3.02% and 1.73%, respectively), *Ruminococcus* 2 (2.70% and 1.39%, respectively), *Phascolarctobacterium* (2.39% and 2.77%, respectively) and *Ruminococcus* 1 (2.06% and 2.45%, respectively); and b) Bacteroidetes phylum, *Bacteroides* (14.45% and 17.82%, respectively), *Prevotella* 9 (7.77% and 8.71%, respectively) and *Parabacteroides* (2.08% and 2.05%, respectively). No significant difference in the main genera were found between the groups.

The diversity measurements obtained for the overweight/obese and lean groups at the ASV level showed non-significant differences, which indicated that the overweight/obese group had a higher Shannon diversity index (*p* = 0.066) (Figure 1b). Moreover, the microbiota richness results based on the Chao1 richness estimator showed a statistically significant difference between the groups (*p* = 0.033), which indicated that the richness of the gut microbiota was significantly lower in the overweight/obese group than in the lean group (Figure 1c). Thus, the microbiota in the overweight/obese group presented fewer bacterial taxa but higher species evenness. Finally, to assess the distribution and variability in the microbiota profiles between the two groups, we performed a PCoA test, and a significant difference in the gut microbiota structure was detected between the two groups (PERMANOVA *p* = 0.00166) (Figure 1d).

**Figure 2:**
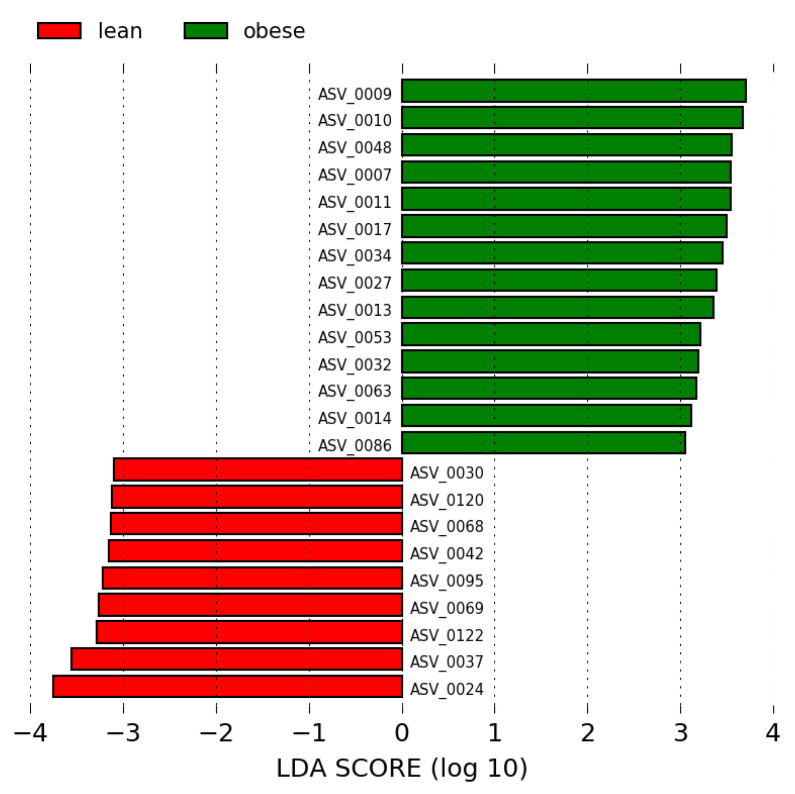
LEfSe analysis between the overweight/obese (**green**) and lean (**red**) groups (LDA score > 3.0). The LDA score (log10) for the most prevalent ASV in the overweight/obese group is represented on a positive scale and the LDA score for the most prevalent ASV in the lean group is represented on a negative scale.

We performed a LEfSe analysis of the overweight/obese and lean groups using LDA scores > 2.5 and >3.0. A total of 23 ASV biomarkers showed significantly different abundances between the two groups with LDA scores > 3. In this case, 14 ASV biomarkers were increased in the overweight/obese group, and 19 ASV biomarkers were increased in the lean group, as shown in Figure 2 and Supplementary Table S1.

Of the 14 biomarkers in the overweight/obese group, 10, two and two belonged to Firmicutes, Actinobacteria and Bacteroidetes, respectively. In the Firmicutes phylum, the families Lachnospiraceae (*Dorea*, *Blautia*, *Coprococcus*, *Lachnospiraceae*_ND3007_group, *Fusicatenibacter* and *Agathobacter*) and Ruminococcaceae (*Faecalibacterium* and *Subdoligranulum*) exhibited the highest abundance in the obese group. Coriobacteriaceae (*Collinsella*) and Bifidobacteriaceae (*Bifidobacterium*), which belong to the Actinobacteria phylum and Bacteroidaceae (*Bacteroides*) and Prevotellaceae (*Prevotella* NK3B31) in the Bacteroidetes phylum were the most enriched families in this group. Principally, the three ASV biomarkers in the overweight/obese group with the most discrimination power and the highest LDA score > 3 were ASVs 0010, 0009 and 0048, which belong to the species *Dorea longicatena*, *Collinsella aerofaciens* and *Bacteroides plebeius*, respectively.

In contrast, 17, 13 and one of the 31 biomarkers detected for the lean group belonged to Firmicutes (Ruminococcaceae, Lachnospiraceae, Christensenellaceae and Veillonellaceae), Bacteroidetes (Bacteroidaceae, Prevotellaceae, Tannerellaceae and Marinifilaceae) and Tenericutes (Anaeroplasmataceae), respectively. The four ASV biomarkers in the lean group with the most discrimination power and highest LDA score > 3 were ASV0024, ASV0037, ASV0122 and ASV0069, and these were identified as *Faecalibacterium prausnitzii*, *Lachnospiraceae* NK4A136 group, *Prevotella* 9 and *Christensenellaceae* R-7 group, respectively.

#### 3.3.1. Associations between Clinical Variables and the Gut Microbiota

**Figure 3:**
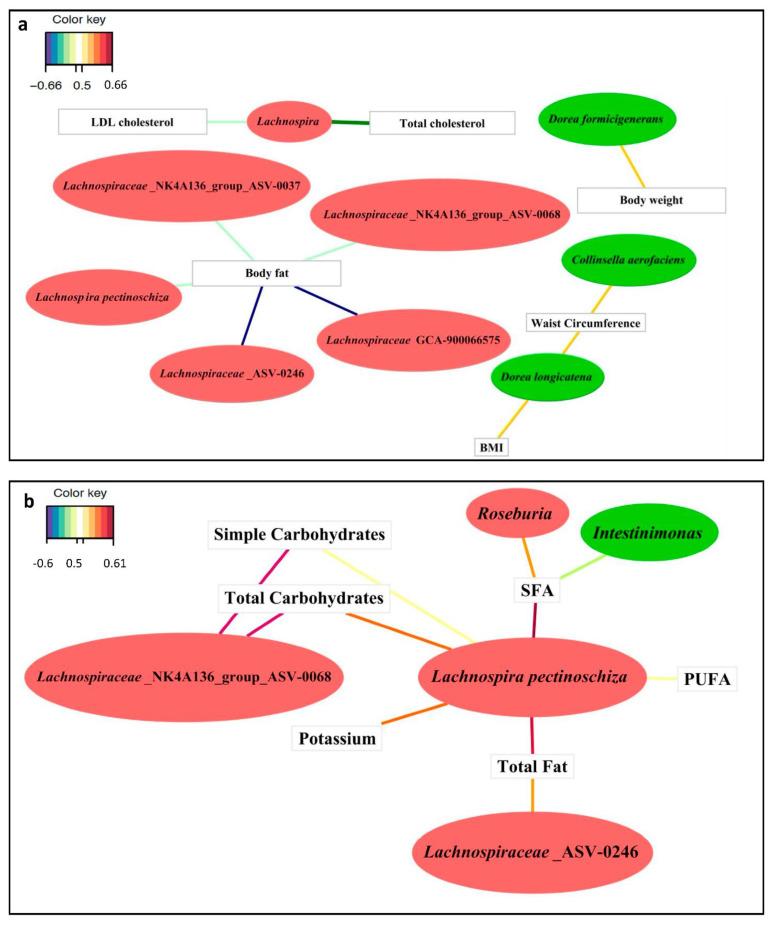
Relevant associations at the ASV level detected by sPLS. (**a**) Relevant correlations for anthropometric variables and the lipid profile with the ASVs showing the greatest difference between the overweight/obese and lean groups. (**b**) Relevant associations between dietary intake variables and the ASVs showing the greatest difference between the overweight/obese and lean groups. Green bubbles show ASVs biomarkers that were significantly increased in the overweight/obese group, and red bubbles show ASVs biomarkers that were significantly increased in the lean group; According to Color key code, the positive and negative associations were related with line colors: purple, red, orange and yellow colors indicated positive associations (from more to less correlation index); blue and green colors indicated negative associations (from more to less correlation index).

We assessed the associations between ASVs with differential abundance and clinical variables through a multivariable sPLS analysis. Three biomarkers, *Collinsella aerofaciens*, *Dorea longicatena* and *Dorea formicigenerans*, which were more abundant in the overweight/obese group, presented positive associations with anthropometric variables: (a) WC exhibited positive associations with the abundance of *Collinsella aerofaciens* and *Dorea longicatena*; (b) BMI showed a positive association with *Dorea longicatena*; and (c) the body weight was positively associated with *Dorea formicigenerans* (Figure 3a). Body fat was the only anthropometric variable with a negative association with five biomarkers of the lean group: *Lachnospiraceae* NK4A136 group (ASV0037), *Lachnospiraceae* NK4A136 group (ASV0068), *Lachnospiraceae* GCA-900066575, a member of the Lachnospiraceae family (ASV0246) and *Lachnospira pectinoschiza* (Figure 3a).

Additionally, the genus *Lachnospira*, which was found at a higher abundance in the lean group than in the overweight/obese group, also showed negative associations with the total cholesterol and LDL cholesterol levels (Figure 3a).

No associations were found between the gut microbiota and the following clinical variables: glucose, HDL cholesterol, triglycerides, SBP and DBP.

#### 3.3.2. Associations between Diet and the Gut Microbiota

We determined the associations between differentially abundant ASVs and energy and nutrient intake data through an sPLS analysis with an association index > 0.5 (Figure 3b). SFAs were negatively associated with *Intestinimonas*, a genus that was significantly more abundant in the overweight/obese group, and SFAs were also positively associated with the genus *Roseburia*, an ASV biomarker with increased abundance in the lean group (Figure 3b). The total fat intake was positively associated with a member of Lachnospiraceae (ASV0246) and *Lachnospira pectinoschiza*, which are biomarkers of the lean group (Figure 3b). Moreover, simple carbohydrates and total carbohydrates were positively correlated with the *Lachnospiraceae* NK4A136 group (ASV0068) and *Lachnospira pectinoschiza*, both of which exhibited higher abundance in the lean group (Figure 3b). The intake of total fat, SFAs, PUFAs, total and simple carbohydrates and potassium were positively associated with the species *Lachnospira pectinoschiza*, an ASV biomarker with increased abundance in the lean group, and this species exhibits a stronger relationship with diet, as determined through an sPLS analysis (|Correlation Index r| > 0.5) (Figure 3b).

## 4. Discussion

To the best of our knowledge, the present cross-sectional study provides new insight into the relationships between clinical variables and dietary intake and the gut microbiota in overweight/obese and lean subjects.

The first studies on the intestinal microbiome and obesity were performed in mice, and the results indicated an important role for the microbiota in obesity, as demonstrated by the finding that obese mice exhibit a lower diversity and a higher ratio of the relative abundance of Firmicutes to that of Bacteroidetes than lean mice [22]. However, human cohort studies have yielded conflicting results. The present study revealed that the ratio of Firmicutes to Bacteroidetes was higher in the overweight/obese group than in the lean group. These results are consistent with those obtained by other researchers, who found that the gut microbiota of lean subjects showed higher levels of Bacteroidetes and lower levels of Firmicutes and detected the opposite results in obese subjects [6]. In fact, different studies have proposed that Firmicutes would be more effective in extracting energy and absorbing calories from food, which would induce subsequent body weight gain [6,22]. Otherwise, data from the present study revealed that the gut microbiota of the lean group exhibited a higher richness than that of the overweight/obese group. Our results are consistent with those obtained in another study that found that obese subjects exhibit a lower bacterial richness than non-obese subjects [23]. In agreement with our data, a cross-sectional study revealed that the overweight/obese group presented greater diversity than the lean group [24]. Thus, the lower richness and the greater diversity found in overweight/obese individuals would suggest a markedly more homogeneous microbiota, based on Shannon index results.

With the aim of identifying the distinctive differences between lean and overweight/obese groups, the present work found that the overweight/obese group exhibited depletion of butyrate-producing bacteria, such as *Faecalibacterium prausnitzii* and *Lachnospiraceae* NK4A136 group [25]. In particular, the species *Faecalibacterium prausnitzii* has been described as a protective species against obesity with anti-inflammatory effects but is abundantly found in the gut of lean subjects [26]. Interestingly, the present study provides the first identification of the genus *Lachnospiraceae* NK4A136 as an ASV biomarker of lean status in humans. Accordingly, a study in mice demonstrated that the *Lachnospiraceae* NK4A136 genus exerts protective and anti-inflammatory effects as a potential butyrate producer [8,25].

In contrast, the species belonging to the Firmicutes phylum that produce butyric acid was replaced in the overweight/obese group by other genera belonging to the same phylum, such as *Dorea*, *Blautia*, *Coprococcus* or *Subdoligranulum*, which could yield other proportions of butyrate, propionate and acetate. Moreover, *Collinsella aerofaciens* and *Bifidobacterium*, which have been described as fibre degraders and H2 consumers and produce mainly lactate and acetate, were also increased in the overweight/obese group [27]. Moreover, *Bacteroides plebeius*, which is also a discriminant species for the overweight/obese group, has also been described as a producer of propionate and acetate [27].

Various studies conducted in recent years have noted that increased acetate exerts an inducer effect on obesity because acetic acid stimulates the synthesis of fatty acids and cholesterol as well as fat storage [6].

Interestingly, we showed that the LDL and total cholesterol levels were higher in the overweight/obese group than in the lean group and that overweight/obese individuals presented a lower abundance of the *Lachnospira* genus than the lean group. Moreover, the *Lachnospira* genus is a short-chain fatty acid (SCFA) producer [28], and a reduction in the *Lachnospira* abundance accompanied by weight gain has been described in overweight adults [28]. Additionally, the *Lachnospira* genus exhibited a negative association with fasting blood glucose in diabetic rat models [29]. For the first time, our work negatively related the *Lachnospira* genus with the serum LDL-C concentration, and our results agreed with the finding that a reduction in the abundance of *Lachnospira* is associated with increases in cardiovascular disease risk factors.

In addition to the main differences between the lean and overweight/obese groups, the present work also found a strong negative association between body fat and the family Lachnospiraceae and found a relationship between this bacterial family and a lean status. Contrary to our results, a study with human stool from Ghanaian volunteers showed that lean subjects exhibited a lower abundance of the family Lachnospiraceae than obese subjects [30]. Additionally, a positive association has been found between the family Lachnospiraceae and metabolic disorders, such as obesity [31]. These contradictory associations might have been obtained be because different species belonging to the same bacterial genus could play distinct roles in the complex context of obesity.

Moreover, *Collinsella aerofaciens*, *Dorea formicigenerans* and *Dorea longicatena*, which are biomarkers of overweight/obese subjects, were positively associated with body weight, WC and BMI. Consequently, as has been proposed, one of the main discriminant ASV biomarkers for obesity is the species *Collinsella aerofaciens* [32]. Supporting our results, *Dorea longicatena* was previously found to be significantly increased in obese subjects [33]. Thus, the present study not only described the abundance of ASV biomarkers in obese subjects but also confirmed the association between anthropometric parameters and ASV biomarkers.

The degradation of polysaccharides and the fermentation of simple carbohydrates by gut microbiota have been extensively studied, but the knowledge on the involvement of intestinal bacteria in fatty acid metabolism remains scarce. Interestingly, the SFA, total fat and PUFA intake showed positive correlations with the Lachnospiraceae family, specifically *Roseburia* and *Lachnospira*, which are genera that were found to be increased in the lean group. In contrast, the SFA intake was negatively associated with *Intestinimonas*, a genus that was increased in the overweight/obese group. Recent studies have revealed that the health effects of foods cannot be predicted by their content of any nutrient group, such as SFAs, without considering the overall macronutrient distribution. Different foods relatively rich in SFAs, such as whole-fat dairy, unprocessed meat and dark chocolate, have different complex matrices that are not associated with an increased risk of cardiovascular disease [34]. Thus, our results might be explained by the consumption of SFAs not related to an increased cardiovascular disease risk. Based on our results, the genus *Roseburia* (belonging to the Lachnospiraceae family) is a propionate and butyrate producer that increases the energy expenditure, which suggests an influence on reducing body weight. Moreover, the *Roseburia* genus is also related to the maintenance of gut health and the immune system, for example, the homeostasis of T-cells and the production of SCFAs, and exhibits anti-inflammatory properties [35]. Thus, the positive correlation between the intake of SFAs and the genus *Roseburia* can be explained by the fact that SFA micronutrients can increase the bacterial community of a genus related to gut health and lean status.

Similar to our results, the *Intestinimonas* genus, which is also a butyrate producer, exhibits a reduced abundance in adult obese subjects [36]. Moreover, *Intestinimonas* from a faecal sample can convert lysine into butyrate and acetate when grown in synthetic medium, which suggests that dietary protein could be a source of butyrate in the human colon, and its conversion by butyrogenic bacteria such as *Intestinimonas* might protect the host from undesired metabolites [37].

To the best of our knowledge, this study provides the first demonstration of an inverse relationship between the *Intestinimonas* genus and SFA consumption in overweight/obese subjects. This new dietary-gut microbiota association has to be confirmed in future RCTs.

Moreover, our results showed that the consumption of total fat, total and simple carbohydrates and potassium exhibited a positive association with the family Lachnospiraceae ASV 0246 and ASV 0037, which are ASV biomarkers that exhibit an increased abundance in the lean group. Additionally, PUFAs, SFAs, total fat and simple carbohydrates and potassium, among other markers, were significantly positively correlated with *Lachnospira pectinoschiza* spp., a species that exhibited an increased abundance in the lean group. In line with our results, a cross-sectional study of lean subjects that evaluated the associations of fat intake (validated by a 131-item semiquantitative Food Frequency Questionnaire) and the gut microbiota revealed a positive association between 21 different Lachnospiraceae species and n-3 PUFA consumption [38]. Moreover, the effects of diet on gut bacteria showed that the consumption of sugar increased the abundance of the family Lachnospiraceae in rat models, independent of the BMI status [39].

The results of the present study highlight a significantly positive relationship for the *Lachnospiraceae* genera and particularly the species *Lachnospira pectinoschiza* with dietary fat and carbohydrates in lean subjects, which suggests that diet influences the abundance of *Lachnospira* and supports the lean characteristics of the subjects. Thus, the present work provides a possible novel strategy for an interaction between diet (dietary fat and carbohydrates) and gut microbiota by increasing the species *Lachnospira pectinoschiza* and the lean body weight status. This new dietary strategy will have to be verified in future RCTs.

Curiously, after assessing all the associations, the Lachnospiraceae family appears to exhibit a link between dietary intake and clinical variables. As mentioned above, the Lachnospiraceae family includes many different genera and bacteria, and thus, the authors can more easily identify this relationship than draw accurate conclusions.

Some of the strengths of this study are that used a multivariant model (sPLS) that considers more variables, such as the gut microbiota, which is composed of many bacteria interacting with each other, than Spearman correlations, which only analyzed the relationship of one variable with another. As a result, to our knowledge, we showed for the first time a new association between clinical variables and the gut microbiota.

The present work has some limitations: the sample size of the study was small, and the observational design of the study does not allow studying the causal interference of the presented dietary results. The dietary intake extraction of the results has been evaluated using the most accurate method (3-day dietary record), providing dietary intake information about the 3 days prior to biological sample collection (fecal and blood) of each subject. Thus, 3 days is a short and closely time in relationship with fecal microbiota results, due that gut microbiota is sensible to diet and can rapidly change [40]. However the associations related to obesity-gut microbiota-diet can be related to the results of diet variations, and the results in this regard have to be taken with caution.

## 5. Conclusions

In conclusion, the present work provides important associations for the gut microbiota with clinical variables and dietary intake. Body weight, WC and BMI showed a positive association with *Dorea formicigenerans*, *Dorea longicatena* and *Collinsella aerofaciens*, which were identified as ASV biomarkers with increased abundances in the overweight/obese group and are species that could be considered microbiota biomarkers of obesity. A negative association was observed between body fat or the LDL or total cholesterol levels and the Lachnospiraceae family, mainly *Lachnospira* pectinoschiza, which suggests that a reduction in the abundance of *Lachnospira* increases lipid cardiovascular disease risk factors. Differing from the results obtained for the subject BMI, the consumption of SFAs was found to be associated with bacterial butyrate producers, negatively associated with the genus *Intestinimonas* an ASV biomarkers in the overweight/obese group and positively associated with the genus *Roseburia* an ASV biomarkers in the lean group. Thus, the relationship between diet and the gut microbiota opens new opportunities for the management of obesity. Moreover, the results of the present work have to be taken with caution as they are associations obtained through an observational study and cannot show effects or mechanisms of action because RCTs would be needed. Further studies are required to confirm the present results.

## Figures and Tables

**Table 1:** General characteristics of overweight/obese and lean subjects included in the cross-sectional study.

Characteristics	Overweight and Obese Subjects (*n* = 96)	Lean Subjects (*n* = 32)	*p* Value
Age, y	52.2 ± 9.7	40.2 ± 8.9	<0.001
Female, %	38.5	50.0	0.254
Systolic blood pressure, mm Hg	129.8 ± 15.9	109.7 ± 7.1	<0.001
Diastolic blood pressure, mm Hg	81.1 ± 9.6	65.8 ± 5.9	<0.001
Pulse pressure, mm Hg	68.4 ± 8.8	43.8 ± 6.8	<0.001
Physical Activity, %			0.180
Inactive	7.3	0.0	
Very low activity	3.1	10.0	
Low activity	14.6	6.7	
Moderate activity	20.8	20.0	
High activity	54.2	63.3	
Anthropometric parameters			
Body mass index, kg/m^2^	31.2 ± 3.4	23.9 ± 2.6	<0.001
Waist circumference, cm			
Male	111.2 ± 8.1	87.7 ± 6.6	<0.001
Female	99.3 ± 7.6	80.5 ± 9.7	<0.001
Fat mass, %	34.9 ± 8.3	22.3 ± 8.1	<0.001
Fat mass, kg	30.6 ± 7.8	14.9 ± 6.3	<0.001
Lean mass, kg	58.0 ± 13.8	53.6 ± 11.0	0.146
Muscle mass, kg	55.1 ± 13.1	50.9 ± 10.5	0.157
Bone mass, kg	2.8 ± 0.6	2.6 ± 0.5	0.162
Total water, %	46.6 ± 6.4	55.1 ± 8.5	<0.001
Total water, kg	41.8 ± 10.3	37.8 ± 8.7	0.073
Blood parameters			
Fasting glucose, mg/dl	94.5 ± 10.2	80.3 ± 7.2	<0.001
Total cholesterol, mg/dl	205.1 ± 30.8	179.6 ± 34.6	<0.001
LDL-cholesterol, mg/dl	127.4 ± 26.3	99.4 ± 33.5	<0.001
HDL-cholesterol, mg/dl	52.7 ± 13.0	63.1 ± 17.6	0.001
Triglycerides, mg/dl	125.3 ± 56.8	85.5 ± 43.0	<0.001

Abbreviations: LDL, low-density lipoprotein; HDL, high-density lipoprotein. Data are mean ± SD (standard deviation), unless otherwise indicated.

**Table 2:** Characteristics of dietary intake of overweight/obese and lean subjects included in the cross-sectional study.

Energy, Macro- and Micronutrients	Overweight and Obese Group (*n* = 96)	Lean Group (*n* = 32)	*p* Value
Energy, kcal/day	2055.9 ± 604.3	2095.9 ± 502.8	0.184
CHO, % energy	36.0 ± 6.3	39.9 ± 6.8	0.013
CHO, grams	181.2 ± 60.9	206.0 ± 53.1	0.005
Simple CHO, % energy	15.5 ± 4.8	17.5 ± 4.1	0.043
Simple CHO, grams	78.3 ± 35.8	92.1 ± 35.9	0.014
Complex CHO, % energy	20.6 ± 5.7	22.3 ± 5.1	0.301
Complex CHO, grams	103.8 ± 41.9	113.8 ± 24.8	0.017
Protein, % energy	18.1 ± 4.3	17.6 ± 3.3	0.702
Protein, grams	90.07 ± 24.9	90.3 ± 22.9	0.369
Total fat, % energy	41.6 ± 5.6	40.0 ± 7.0	0.472
Total fat, grams	97.4 ± 34.5	95.0 ± 33.1	0.429
SFA, % energy	12.4 ± 2.8	11.0 ± 2.7	0.083
SFA, grams	29.6 ± 13.1	26.5 ± 10.8	0.515
MUFA, % energy	19.0 ± 3.2	19.0 ± 4.5	0.506
MUFA, grams	44.0 ± 15.0	45.3 ± 17.7	0.162
PUFA, % energy	6.6 ± 2.0	6.6 ± 1.8	0.710
PUFA, grams	15.6 ± 7.4	15.6 ± 6.0	0.247
Fibre, g/day	18.8 ± 6.8	23.7 ± 9.1	<0.001
Alcohol, g/day	12.9 ± 18.8	7.4 ± 9.2	0.327
Dietary cholesterol, mg/day	391.8 ± 188.5	341.1 ± 164.4	0.259
Sodium, mg/day	2580.8 ± 928.6	2353.2 ± 728.1	0.485
Potassium, mg/day	3042.4 ± 868.5	3448.3 ± 826.2	0.002
Calcium, mg/day	752.9 ± 354.2	789.3 ± 339.9	0.549
Magnesium, mg/day	309.5 ± 106.9	345.2 ± 74.0	0.001

CHO, carbohydrates; SFA, saturated fatty acids; MUFA, monounsaturated fatty acids; PUFA, polyunsaturated fatty acids. Data expressed as Mean ± standard deviation. Differences in categorical variables were examined using chi-squared analysis and presented as percentage (%). Differences in continuous variables were examined using T-Student for parametric variables and using U the Mann-Whitney for non-parametric variables. Statistical significance was set at *p* Value < 0.05.

## Data Availability

The data presented in this study are available on request from the corresponding author.

## Supplementary Materials

The following are available online at https://www.mdpi.com/article/10.3390/nu13062032/s1, Figure S1: Phylum-level comparison. The significantly differences in the gut microbiota between the overweight/obese and lean groups are shown, Table S1: Significant differences between the overweight/obese and lean groups found for the relative abundance of ASVs that presented LDA scores > 2.5 in the LEfSe analysis.
