# Supplementary material for: Gut Microbiota Profile and Its Association with Clinical Variables and Dietary Intake in Overweight/Obese and Lean Subjects: A Cross-Sectional Study
Source: Nutrients. 2021 Jun 13;13(6):2032. doi: 10.3390/nu13062032 (PMC8231825; doi:10.3390/nu13062032)
Supplement: Supplementary file 1 [file nutrients-13-02032-s001.zip › Supplementary Figures.pptx]

## Slide 1
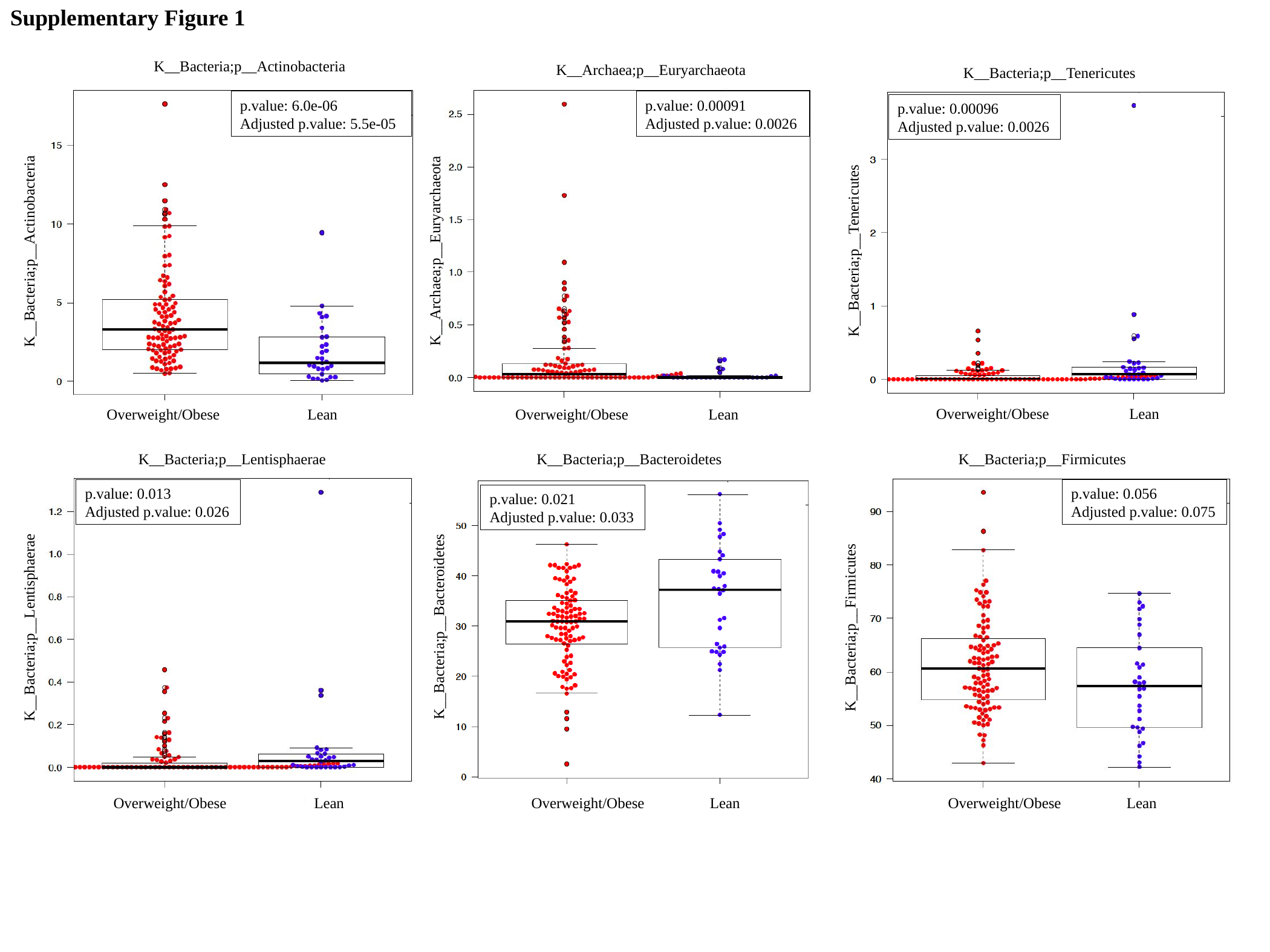

Supplementary Figure 1
K__Bacteria;p__Actinobacteria
K__Archaea;p__Euryarchaeota
K__Bacteria;p__Tenericutes
p.value: 0.00091
Adjusted p.value: 0.0026
p.value: 6.0e-06
Adjusted p.value: 5.5e-05
p.value: 0.00096
Adjusted p.value: 0.0026
K__Bacteria;p__Tenericutes
K__Archaea;p__Euryarchaeota
K__Bacteria;p__Actinobacteria
Overweight/Obese
Lean
Overweight/Obese
Lean
Overweight/Obese
Lean
K__Bacteria;p__Bacteroidetes
K__Bacteria;p__Firmicutes
K__Bacteria;p__Lentisphaerae
p.value: 0.013
Adjusted p.value: 0.026
p.value: 0.056
Adjusted p.value: 0.075
p.value: 0.021
Adjusted p.value: 0.033
K__Bacteria;p__Bacteroidetes
K__Bacteria;p__Firmicutes
K__Bacteria;p__Lentisphaerae
Overweight/Obese
Lean
Overweight/Obese
Lean
Overweight/Obese
Lean
