# Supplementary material for: Gut Microbiota Profile and Its Association with Clinical Variables and Dietary Intake in Overweight/Obese and Lean Subjects: A Cross-Sectional Study
Source: Nutrients. 2021 Jun 13;13(6):2032. doi: 10.3390/nu13062032 (PMC8231825; doi:10.3390/nu13062032)
Supplement: Supplementary file 1 [file nutrients-13-02032-s001.zip › Supplementary Tables.pdf]

Supplementary Table 1. Significant differences between Overweight/Obese and Lean groups in the relative abundance of ASVs that present LDA score > 2.5 in LEfSe analysis.

ASV	LDA score	*P*-value	***Taxonomy***
**Increased in Overweight/Obese Group**			
ASV-0009	3.7010	3.0382e-07	p__Actinobacteria;c__Coriobacteriia;o__Coriobacteriales;f__Coriobacteriaceae;g__Collinsella;s__aerofaciens
ASV-0010	3.6686	2.4853e-09	k__Bacteria;p__Firmicutes;c__Clostridia;o__Clostridiales;f__Lachnospiraceae;g__Dorea;s__longicatena
ASV-0048	3.5504	0.0009	k__Bacteria;p__Bacteroidetes;c__Bacteroidia;o__Bacteroidales;f__Bacteroidaceae;g__Bacteroides;s__plebeius
ASV_0007	3.5402	0.0139	k__Bacteria;p__Firmicutes;c__Clostridia;o__Clostridiales;f__Ruminococcaceae;g__Faecalibacterium;s__NA
ASV-0011	3.5393	0.0041	p__Firmicutes;c__Clostridia;o__Clostridiales;f__Ruminococcaceae;g__Subdoligranulum;s__NA
ASV-0017	3.4943	0.0100	k__Bacteria;p__Actinobacteria;c__Actinobacteria;o__Bifidobacteriales;f__Bifidobacteriaceae;g__Bifidobacterium;s__NA
ASV-0034	3.4552	0.0109	k__Bacteria;p__Bacteroidetes;c__Bacteroidia;o__Bacteroidales;f__Prevotellaceae;g__Prevotellaceae_NK3B31_group;s__NA
ASV-0027	3.3948	7.9115e-05	p__Firmicutes;c__Clostridia;o__Clostridiales;f__Lachnospiraceae;g__Fusicatenibacter;s__saccharivorans
ASV-0013	3.3540	0.0393	p__Firmicutes;c__Clostridia;o__Clostridiales;f__Ruminococcaceae;g__Subdoligranulum;s__variabile
ASV-0053	3.2178	1.8499e-05	p__Firmicutes;c__Clostridia;o__Clostridiales;f__Lachnospiraceae;g__Lachnospiraceae_ND3007_group;s__NA
ASV-0032	3.1956	0.0001	p__Firmicutes;c__Clostridia;o__Clostridiales;f__Lachnospiraceae;g__Coprococcus_3;s__comes
ASV-0063	3.1718	6.6223e-05	p__Firmicutes;c__Clostridia;o__Clostridiales;f__Lachnospiraceae;g__NA;s__NA
ASV-0014	3.1230	0.0178	p__Firmicutes;c__Clostridia;o__Clostridiales;f__Lachnospiraceae;g__Agathobacter;s__NA
ASV-0086	3.0490	5.1063e-06	p__Firmicutes;c__Clostridia;o__Clostridiales;f__Lachnospiraceae;g__Blautia;s__massiliensis
ASV-0065	2.9363	0.0011	p__Firmicutes;c__Clostridia;o__Clostridiales;f__Lachnospiraceae;g__Blautia;s__faecis
ASV-0066	2.9214	0.0002	p__Firmicutes;c__Clostridia;o__Clostridiales;f__Lachnospiraceae;g__Dorea;s__formicigenerans
ASV-0180	2.9176	0.0046	k__Bacteria;p__Bacteroidetes;c__Bacteroidia;o__Bacteroidales;f__Bacteroidaceae;g__Bacteroides;s__plebeius
ASV-0067	2.9152	0.0003	p__Firmicutes;c__Clostridia;o__Clostridiales;f__Lachnospiraceae;g__NA;s__NA
ASV-0127	2.9128	0.0001	p__Firmicutes;c__Clostridia;o__Clostridiales;f__Peptostreptococcaceae;g__Romboutsia;s__NA
ASV-0062	2.9014	0.0154	k__Bacteria;p__Bacteroidetes;c__Bacteroidia;o__Bacteroidales;f__Bacteroidaceae;g__Bacteroides;s__coprocola
ASV-0115	2.8851	0.0015	p__Euryarchaeota;c__Methanobacteria;o__Methanobacteriales;f__Methanobacteriaceae;g__Methanobrevibacter;s__NA
ASV-0197	2.8563	0.0009	p__Firmicutes;c__Clostridia;o__Clostridiales;f__Clostridiaceae_1;g__Clostridium_sensu_stricto_1;s__NA
ASV-0125	2.8216	1.3212e-05	p__Firmicutes;c__Clostridia;o__Clostridiales;f__Lachnospiraceae;g__Agathobacter;s__NA
ASV-0073	2.7978	0.011	p__Firmicutes;c__Clostridia;o__Clostridiales;f__Lachnospiraceae;g__Blautia;s__NA
ASV-0263	2.7777	0.037	k__Bacteria;p__Firmicutes;c__Negativicutes;o__Selenomonadales;f__Acidaminococcaceae;g__Phascolarctobacterium;s__succinatutens
ASV-0149	2.7351	0.0002	p__Firmicutes;c__Clostridia;o__Clostridiales;f__Ruminococcaceae;g__Ruminiclostridium_5;s__NA
ASV-0272	2.6877	0.0057	k__Bacteria;p__Firmicutes;c__Clostridia;o__Clostridiales;f__Clostridiaceae_1;g__Clostridium_sensu_stricto_1;s__NA
ASV-0175	2.6862	0.0002	k__Bacteria;p__Firmicutes;c__Clostridia;o__Clostridiales;f__Ruminococcaceae;g__Negativibacillus;s__NA
ASV-0163	2.6118	0.0073	k__Bacteria;p__Firmicutes;c__Clostridia;o__Clostridiales;f__Lachnospiraceae;g__NA;s__NA
ASV-0189	2.5825	0.0070	k__Bacteria;p__Firmicutes;c__Clostridia;o__Clostridiales;f__Lachnospiraceae;g__CAG-56;s__NA
ASV-0247	2.5788	0.0020	k__Bacteria;p__Actinobacteria;c__Coriobacteriia;o__Coriobacteriales;f__Eggerthellaceae;g__NA;s__NA
ASV-0112	2.5572	0.0137	k__Bacteria;p__Firmicutes;c__Clostridia;o__Clostridiales;f__Ruminococcaceae;g__Ruminococcus_2;s__NA

ASV	LDA score	*P*-value	*Taxonomy*
**Increased in Lean Group**			
ASV-0024	3.7464	0.0463	k__Bacteria;p__Firmicutes;c__Clostridia;o__Clostridiales;f__Ruminococcaceae;g__Faecalibacterium;s__prausnitzii
ASV-0037	3.5586	0.0120	p__Firmicutes;c__Clostridia;o__Clostridiales;f__Lachnospiraceae;g__Lachnospiraceae_NK4A136_group;s_NA
ASV-0122	3.2856	0.0017	k__Bacteria;p__Bacteroidetes;c__Bacteroidia;o__Bacteroidales;f__Prevotellaceae;g__Prevotella_9;s__NA
ASV-0069	3.2612	0.0035	p__Firmicutes;c__Clostridia;o__Clostridiales;f__Christensenellaceae;g__Christensenellaceae_R-7_group;s__NA
ASV-0095	3.2187	0.0039	p__Firmicutes;c__Clostridia;o__Clostridiales;f__Lachnospiraceae;g__Lachnospira;s__pectinoschiza
ASV-0042	3.1557	0.0139	k__Bacteria;p__Firmicutes;c__Clostridia;o__Clostridiales;f__Lachnospiraceae;g__Lachnospira;s__NA
ASV-0068	3.1239	0.0244	p__Firmicutes;c__Clostridia;o__Clostridiales;f__Lachnospiraceae;g__Lachnospiraceae_NK4A136_group;s_NA
ASV-0120	3.1169	0.0007	p__Firmicutes;c__Clostridia;o__Clostridiales;f__Lachnospiraceae;g__Roseburia;s__NA
ASV-0030	3.0980	0.0447	p__Firmicutes;c__Clostridia;o__Clostridiales;f__Ruminococcaceae;g__Ruminococcaceae_UCG-002;s__NA
ASV-0090	2.9625	0.0124	p__Bacteroidetes;c__Bacteroidia;o__Bacteroidales;f__Bacteroidaceae;g__Bacteroides;s__NA
ASV-0148	2.9346	0.0003	p__Firmicutes;c__Clostridia;o__Clostridiales;f__Lachnospiraceae;g__NA;s__NA
ASV-0202	2.9245	0.0009	p__Bacteroidetes;c__Bacteroidia;o__Bacteroidales;f__Bacteroidaceae;g__Bacteroides;s__NA
ASV-0168	2.9224	0.0188	p__Firmicutes;c__Clostridia;o__Clostridiales;f__Lachnospiraceae;g__Lachnospiraceae_UCG-001;s__NA
ASV-0250	2.9157	0.0015	k__Bacteria;p__Bacteroidetes;c__Bacteroidia;o__Bacteroidales;f__Prevotellaceae;g__Prevotella_9;s__NA
ASV-0249	2.9084	0.0463	k__Bacteria;p__Bacteroidetes;c__Bacteroidia;o__Bacteroidales;f__Prevotellaceae;g__Alloprevotella;s__NA
ASV-0132	2.9080	0.0003	p__Firmicutes;c__Clostridia;o__Clostridiales;f__Lachnospiraceae;g__Roseburia;s__hominis
ASV-0229	2.9034	0.0213	k__Bacteria;p__Tenericutes;c__Mollicutes;o__Anaeroplasmatales;f__Anaeroplasmataceae;g__Anaeroplasma;s__NA
ASV-0105	2.8317	0.0445	k__Bacteria;p__Firmicutes;c__Clostridia;o__Clostridiales;f__Lachnospiraceae;g__Coprococcus_2;s__NA
ASV-0385	2.7632	0.0099	k__Bacteria;p__Bacteroidetes;c__Bacteroidia;o__Bacteroidales;f__Prevotellaceae;g__Prevotella_9;s__NA
ASV-0987	2.7305	0.0099	k__Bacteria;p__Bacteroidetes;c__Bacteroidia;o__Bacteroidales;f__Bacteroidaceae;g__Bacteroides;s__NA
ASV-0195	2.7287	0.0403	k__Bacteria;p__Bacteroidetes;c__Bacteroidia;o__Bacteroidales;f__Bacteroidaceae;g__Bacteroides;s__caccae
ASV-0217	2.7060	0.0360	p__Bacteroidetes;c__Bacteroidia;o__Bacteroidales;f__Bacteroidaceae;g__Bacteroides;s__NA
ASV-0190	2.7042	0.0001	p__Bacteroidetes;c__Bacteroidia;o__Bacteroidales;f__Marinifilaceae;g__Odoribacter;s__splanchnicus
ASV-0206	2.6492	0.0286	k__Bacteria;p__Bacteroidetes;c__Bacteroidia;o__Bacteroidales;f__Marinifilaceae;g__Odoribacter;s__splanchnicus
ASV-0459	2.6381	0.0015	k__Bacteria;p__Bacteroidetes;c__Bacteroidia;o__Bacteroidales;f__Prevotellaceae;g__Alloprevotella;s__NA
ASV-0344	2.6365	0.0131	k__Bacteria;p__Firmicutes;c__Clostridia;o__Clostridiales;f__Lachnospiraceae;g__Lachnospiraceae_NK4A136_group;s__NA
ASV-0466	2.5738	0.0099	k__Bacteria;p__Firmicutes;c__Negativicutes;o__Selenomonadales;f__Veillonellaceae;g__Anaerovibrio;s__NA
ASV-0420	2.5711	0.0216	k__Bacteria;p__Bacteroidetes;c__Bacteroidia;o__Bacteroidales;f__Tannerellaceae;g__Parabacteroides;s__distasonis
ASV-0375	2.5255	0.0213	k__Bacteria;p__Firmicutes;c__Clostridia;o__Clostridiales;f__Lachnospiraceae;g__Agathobacter;s__NA
ASV-0754	2.5250	0.0128	k__Bacteria;p__Firmicutes;c__Clostridia;o__Clostridiales;f__Ruminococcaceae;g__Ruminococcaceae_UCG-010;s__NA
ASV-0273	2.5197	0.0096	p__Firmicutes;c__Clostridia;o__Clostridiales;f__Lachnospiraceae;g__NA;s__NA

LDA, linear discriminant analysis; o, obese, L, lean; ASV, amplicon sequence variant.
P-value <0.05 was considered statistically significant.
